# Analysis of antidiabetic, antiulcer and analgesic potential of traditional ethnomedicinal plant *Emex spinosa* (L.) Campd. from Azad Jammu and Kashmir

**DOI:** 10.1371/journal.pone.0274706

**Published:** 2022-10-13

**Authors:** Muhammad Ajaib, Saiqa Ishtiaq, Muhammad Ishtiaq, Mehwish Maqbool, Khizar Hayat Bhatti, Afsheen Khan, Afeera Afreen, Tanveer Hussain, Tauqeer Sardar, Alia Gul, Muhammad Azeem

**Affiliations:** 1 Department of Botany, Mirpur University of Science & Technology (MUST), Mirpur (AJK), Pakistan; 2 University College of Pharmacy, University of the Punjab, Lahore, Pakistan; 3 Department of Botany, University of Gujrat, Gujrat, Pakistan; 4 Department of Botany, Dr. Moinuddin Laboratory of Plant Ecology and Dendrochronology, Federal Urdu University of Arts, Science and Technology, Gulshan e Iqbal, Karachi, Pakistan; 5 Department of Botany, Hazara University, Mansehra, KP, Pakistan; 6 Department of Biology, College of Science, University of Bahrain, Zallaq, Bahrain; King Abdulaziz University, SAUDI ARABIA

## Abstract

In this research antidiabetic, analgesic and antiulcer potential of traditional ethnomedicinal plant: *Emex spinosa* (L.) Campd. (Family Polygonaceae) was evaluated by extracting its phytoconstituents using methanol (MeOH) solvent through maceration protocol. The quantitative phytochemical analysis of the extract revealed flavonoids were highest in leaf extract (15.63±0.93 mg/mL) and with (11.5±0.57 mg/mL) in stem. Alkaloids and tanins were also present in the samples in various conc. while saponins were absent. To confirm pharmaceutical potential of plant against ulcer, diabetes and analgesic infirmities, a model experimental animal wistar albino rats (*Rattus norvegicus*) were used. In antiulcer study, using hot plate method the maximum results were observed with 250 mg/kg in the 2.5 hours of study. The leaf extract showed a 40.41±2.73 latency time and the fruit with a 36.77±2.41, and the stem with a 27.85±3.09, which was comparable to the standard drug Aspirin, *i*.*e*., 47.5±0.57. For analysis of antiulcer potential of the plants parts doses of 250 and 500 mg/kg was applied to check the reclamation of ethanol-induced acute ulcer and of Aspirin-induced chronic ulcer of stomach. In order to confirm efficacy of the drug potential of plant following parameters like microscopic evaluation, gastric volume, total acidity, mucosa weight, ulcer index, pH and histopathology of stomach were analyzed. In antidiabetic analysis, in an acute study after a single dose of 500 mg/kg extract after 2hrs the blood glucose levels were 367±51.09958^NS^, 416±59.79548^NS^, 437.5±61.96437^NS^ mg/dL for leaf, stem and fruit, respectively. After4hrs 351.75±88.27644^NS^ mg/dl, 448.25±25.64948^NS^ mg/dl, 445.25±27.07205^NS^ mg/dl and after 6hrs 354.5±92.70428^NS^, 442±24.60691^NS^, a440±33.16625^NS^ mg/dl, respectively. The analgesic activity was explored by applying hot plate, tail flick and formalin paw licking method. In hot plate method the maximum results were observed with 250mg/kg in the 2.5 hours of study. The leaf extract showed a 40.41±2.73 latency time and the fruit with a 36.77±2.41 and the stem with a 27.85±3.09, which was comparable to the standard drug Aspirin, *i*.*e*., 47.5±0.57. The respective plant extracts at 250mg/kg showed a gradual rise in latency time when compared to the control. It was concluded that all three components of *E*. *spinosa* perform proved to be significant as potential source of herbal medicines to cure different prevalently occurring diseases. Furthermore, it was confirmed through results analysis that plant t can be used to discover novel drug using dedicated high throughput techniques and ethnopharmacological approaches.

## Introduction

Plants have been part and parcel of humanity for long times, probably from the time of emergence of human life on this planet. According one estimate is reported that around 35000–70000 plant species are known as medicinal species to treat various ailments across the world [1]. Plants have been utilized for health, food, shelter, fodder, forage and botanic medicines for thousands of years by the mankind. The herbal drugs or botanic drugs (BDs) provide an opportunity to manufacture modern allopathic medications from different plants for curing acute and chronic diseases [2]. Plants have been used by humans as a natural product of remedies and cures for various infirmities since decades. Herb-based medicines have received special attention among diverse ethnic groups in developing and developed countries since they are often used because botanic drugs are considered as cheap, with less negative effects and possessing more curing impact due to synergetic phenomenon [3]. Plants’ dynamic variety contributes around 12,000 phytochemical compounds which is less than 10% of the overall quantity of chemical compounds. A variety of medicines, most of which are obtained from plants and used in different allopathic medicines combat diseases and other therapies across the world. Around three-quarters of the world’s population receives primary health care facility from plant-based medications or botanic drugs sometimes also called as green medicines (GMs) [4]. Different chemical phytochemical like alkaloids, flavonoids, terpenoids, glycosides, glycerol, tannins, pholabatannis, saponins and steroids are thought to be responsible for plants’ medicinal effect and these phytoconstituents can be harvested to produce or make neo drugs for prompt cure of various fatal infirmities [5].

Man has been facing various disease and Diabetes mellitus is one of these commonly prevailing disorder or infirmity ubiquitously present all around the globe and it has become a severe public health issue of the time. It’s a long-term condition caused by excessive starch, fat and protein in which pancreases becomes functionless or retarded in its production of insulin (hormone). This is caused by a faulty or ineffective insulin secretory reaction or its efficacy to metabolize the carbohydrates. This leads to decreased glucose utilization which is a hallmark of diabetes mellitus, resulting in hyperglycemia with name of high sugar level in blood. Increased osmotic pressure and dehydration of tissue cells was caused by elevated blood glucose levels because glucose doesn’t quite readily transfer through the pores of the cell membrane [6].

Analgesics, also recognized as anesthetics (painkillers), are constituents that act in a variety of techniques to reduce various forms of discomfort or pains in the body. Pain is a frequent sign that suggests that something is abnormal with our body and can be an indication of illness. As a result, it has been established that pain is a very peculiar unpleasant sensation that is independent of the five senses, relying more on its own central and peripheral mechanisms [7]. Pain can be interpreted in a variety of ways as the only symptom of a variety of illnesses is also pain. Nonsteroidal anti-inflammatory medications (NSAIDs) and opioids are used to manage mild to moderate and extreme pains, respectively. Because of their adverse effects, such as stomach irritation, dependence and resistance, medications have certain significant limitations. As a result, there is a need to ramp up research with the goal of producing active agents with low toxicity levels which can only be obtained and produced from the plants [8].

Gastric ulcer, one of the most common problems found in almost all adults around the world particularly who are addictor of coffee, tea and wine or sometime eat spicy foods [9]. Acid, pepsin, bile acids, bacterial products (Helicobacter pylori) and drugs continuously exposed to the gastric mucosa raises the risk of gastric ulcers [10]. Increased stomach acid and pepsin secretion, suppression of prostaglandin synthesis and cell proliferative growth and decreased blood flow and gastrointestinal motility have all been linked to the pathogenesis of gastric ulcer [11]. Peptic ulcers are caused by gastric acid or pepsin destroying a region of the gastrointestinal tract (GIT). A stomach ulcer, oesophageal, or duodenal ulcer are all examples of peptic ulcers. Peptic ulcer symptoms include sickness, eject, swelling, changes in hungriness, and a nagging or flaming sensation. After eating, these sensations usually fade away, only to reemerge later. Duodenal ulcers are more common than stomach ulcers; gastric ulcers account for 15% of cases, whereas duodenal ulcers account for 85%. Although peptic ulcers are uncommon, they are becoming more virulent, which may be due to the global population’s growing age. Peptic ulcers usually appear as a single wound; however, they can also appear in numerous forms [12, 13].

The plant *Emex spinosa* (L.) Cambd. belongs to family Polygonaceae which has two representative members named: *Emex spinosa* (L.) Campd. and *Emex australis* Steinh. The former plant is usually known as spiny emex or three-lobed jack, then modified as little jack or devil’s thorn [14]. *E*. *spinosa* is naturally heteromorphic and give rise to underground and aerial achenes in different areas of AJK and Pakistan. Vegetative structures are less than reproductive structures. Two types of achenes are the characteristic features which make in it a weedy plant [15]. The genus name *Emex* originated from *Rumex*, the genus in which it was originally placed and the Latin word ex, ’out of’ Its species name, *’*spinosa*’* is Latin for ’prickl or thorny’ and apparently refers to its achenes [16]. The plant *E*. *spinosa* (L.) Campd., found in the form of small jack like, vernacularly termed as DIRS- EL-AGOOZ. Plant of this family Polygonaceae occurs near railway tracks and alongside the bank of drains, waste sandy places, and the area where the winter crops will be grown [17].

*Emex spinosa* is considered as a small weed that provides fuel for local residents, serves as a source of food for animals, and may have therapeutic properties. *Emex spinosa* is considered a small weed that provides fuel for local residents, serves as a source of food for animals, and may have therapeutic properties [18]. Its most significant plant which is used as cure for indigestion; increase hunger, it is also used as treatment of stomach ailments. It’s understood that *Emex spinosa* is purgative and diuretic. It is commonly consider it as medicinal plant which will be used to treat in digestion and stomach disorder [19]. African peoples boiled the leaves of *Emex spinosa* plant and then used for relief against dyspepsia and sickness and to stimulate appetite [20]. The leaves of *E*. *spinosa*, as well as its carrot-like tap root, are eaten by locals and Tribes in eastern Saudi Arabia [21]. *Emex spinosa* was recognized as a capable fodder plant in a comparative evaluation of its nutritional properties [22]. The presence of bioactive substances such as phenolic compounds, flavonoids, tannins, and terpenoids in these plants contributes to their potential [23]. *E*. *spinosa* has long been utilized in herbal medicine for its anti-inflammatory and pain-relieving properties [24]. The key problem statement of the research proposal was to explore the green medicines for cure of ulcer, analgesic, diabetes and other chronic disorders in indigenous people of Mirpur division of AJK. Prior to this no pharmacology work is conducted to find the active phytoconstituents for exploration medicinal worth of this wild indigenous plants.

The key objectives of the current study were multifarious including: (i) to explore ethnomedicinal and phytochemical analysis of different parts of the selected plant species: *Emex spinosa* (L.) Campd., (ii) to explore its antidiabetic efficacy, (iii) to explore its analgesic property to cure pains and (iv) to discover and testify its anti-ulcer potential of various parts to be used as herbal drug. This research study will provide promising outcomes for novel drug discovery and drug development from this reknown ethnomedicinal plants which is promulgated in various traditional ethnomedicines (TEMs) in the world.

## Materials and methods

### Collection of plant samples

For analysis, heathy samples of leaf, fruit and stem were meticulously separated from the plant (*Emex spinosa*) were collected in the months of April and May from different villages of Mirpur, AJK. The collected plant parts were shadow dried in the room and powdered for further experimental trials. The plant sample was collected, identified, pressed, dried, mounted on herbarium sheet and submitted in Herbarium (MUH) of Department of Botany, (MUST) Bhimber Campus with voucher specimen number: MUH-316 for future reference studies.

### Preparation of methanolic extract of plant

The dried powdered form of different parts of the plant was extracted in methanol (MeOH) solvent using maceration protocol. About 250 g of powdered plant material was dipped in methanol for seven (07) days, kept shaking by using electrical shaker and sometimes vigorously stirred for though mixing. After seven days, the macerated materials was filtered using Whatman filter paper No.42 and its filtrate was dried using rotary evaporator to yield a sticky concentrated residue for further analysis. All the residue of three parts was kept separately in air tight bottles for future experimental trials.

### Phytochemical analysis

The phytochemical study was carried out using a qualitative analysis by following protocol of Sonam et al., [25]. By using a standard method, alkaloids, flavonoids, carbohydrates, sterols, cardiac glycosides, triterpenoids, saponins, tannins, proteins, and lipids were determined in plant extract. The Harborne procedure was used for quantitative phytochemical analysis of alkaloids, flavonoids, phenols, saponins, and tannins [26].

### Experimental animals

In the study, a total of 40 *Rattus norvegicus* (Wistar albino) rats (weight 180–300 gm), 50 rats of weight (150–250 g) and 40 adult albino mice (23–28 g) of both sexes were obtained from the animal house at the Pharmacy Department of Punjab University in Lahore. Prior to the start of the research, rats were adopted for a week to assure that they were handled properly. All animals were kept in an animal house at a controlled temperature 22±1°C under 12 hrs dark and light cycles. All the animals had free access to food and water ad libitum.

### Ethics declaration

We confirm that all the research meets ethical guidelines and adheres to the legal requirements of the University and our country. The University Institutional Ethics Review Board on request of Departmental ethical committee of the Punjab University has approved the experimental plan and allowed to proceed report vide letter No. D 109/ FIMS. The animals were used as per protocol and procedure Associated Guidelines and SOPs can be found on the ACC SOP/Guidelines website: http://ors.ubc.ca/contents/animal‐care‐sops‐guidelines. During the experiment, the temperature was set to standard environmental laboratory conditions (25 ±5°C) and relative humidity (50–60%).

### Antidiabetic experimental trials

#### Experimental design

The rats (white albino) were divided into seven classes, each consisting of four rats and details of each group constituency is as:

Group-I: They were called the normal control (Nc). There is no Alloxan induction or plant extract induction.

Group-II: Diabetic control group (Dc). This group of rats received alloxan instead of plant extract treatment.

Group-III: This category is referred to as the "treatment control group" (Tc). These rats were injected with Alloxan and treated with Glibenclamid, (10 mg/kg body weight), an allopathic drug available on the market.

Group-IV: This category is thought to be a treatment group (T_1_). These rats were injected with alloxan and given an oral dosage of (500 mg/kg body weight) methanolic extract of *Emex spinosa* leaf per day.

Group-V: This category is thought to be a second treatment group (T_2_). These rats were injected with alloxan and given an oral dosage of (500 mg/kg body weight) methanolic extract of *Emex spinosa* fruit per day.

Group-VI: This is considered to be a third treatment type (T_3_). These rats were given an alloxan injection and a daily oral dose of (500 mg/kg body weight) methanolic extract of *Emex spinosa* stem.

### Induction of diabetes in experimental rats

#### The dose optimization

In order to make the rats diabetic, Alloxan (50–250 mg/kg) was used and its dose was given initially. The experimental animals were divided into four classes. The first group received 50 mg/kg and the second 75 mg/kg. The third got 120 mg/kg, while the fourth got 250 mg/kg. The first group of rats did not get hyperglycemia. Only one rat in the 120 mg group developed diabetes. The fourth group of rats perished after receiving 250 mg/kg of Alloxan. The third group of rats had 120 mg/kg Alloxan which worked well. This dose was optimized using previously established [27] and it was confirmed that 120 mg/kg was optimal dose.

#### Processing of inducing alloxan

A single intraperitoneal (i.p.) injection was given to rats weighing 160–300 g. The preparation of Alloxan monohydrate (120 mg/kg body weight) in ice cold water was performed by pricking a needle into the veins of tail rats and checking their blood glucose level using glucometer prior to intraperitoneal injection. In order to prevent hypoglycemia; after 4 hours of alloxan each rat was given a 2 mL solution of a readily available five percent (5%) glucose solution. The Alloxan dose was chosen with consideration of the rats’ weight and health. Application of three days of provision of injections induced the diabetes. Glucose levels were measured using a glucometer and tail glucose strips. The study used rats with blood glucose level between 250 and 367 mg/dL and the procedure followed was as given by Al-Awadi *et al*., [28]. After making the rabbits diabetic; they were orally administered 500 mg/kg *Emex spinosa* leaf, fruit, and stem extract. Then blood glucose level were measured at 0, 2, 4 and 6 hours interval on daily basis upto seven consecutive days following previous protocol [29].

#### Oral glucose tolerance test

For the oral glucose tolerance test, four groups of rats were formed and each group consisted of four rats with following constituents:

Group A = Received vehicle (normal saline),

Group B = Standard drug (Acarbose 4.28 mg/kg body weight)

Group C = Diabetic group

Group D = treatment control group (TC) given the *E*. *spinosa* fruit extract (500 mg/kg + Glucose (2 g/kg body weight)).

To assess the impact of different extracts on blood glucose level, a procedure of Ajaib et al [30] was used. The blood glucose was measured after every 30 minutes, 1 hour, 1.5 hours, 2 hours, 3 hours, 4 hours, 5 hours, and 6 hours using glucometer (model Accu-Chek Active (Model GU).

#### Analgesic activity analysis (AAA)

Analgesic activity of leaf, fruit, and stem of *E*. *spinosa* were investigated by applying three different methods.

*(i) Hot plate method*. The hot plate method was used to investigate analgesic activity in mice [31]. Thirty mice of either sex was divided into five groups (n = 6). Each group was given one dose of the leaf, fruit and stem extract at rate of 250 mg/kg and 300 mg/kg of aspirin (standard medication) and a control. Animals were lowered onto the surface of a hot plate at temperature of 502°C surrounded by a big beaker and after 30 minutes of extract dose oral engulfing time was started to count. Reaction time (RT) was measured by the time it took the mice to lift or lick their hind limbs (RT). To avoid mice being burned, don’t go above 90 minutes following the protocol of previous research [31].

*(ii) Tail flick method*. The mice were checked for sensitivity by gently submerging their tails in hot water maintained at 55° C for 2–3 cm. The mice who lifted their tails from hot water in less than 5 seconds were chosen for the experiment. Mice that had been starved overnight were put into five groups (n = 6). Each group got 250 mg/kg of leaf, fruit, and stem extract, as well as 300 mg/kg of aspirin, with one group serving as a control. Every 30 minutes, 9–10 readings were taken as previously used protocol [32].

*(iii) Formalin induced paw licking method*. It was a one-day experiment in which overnight starved mice were given an oral dose of extracts or medicine before being injected with 20 microliters formalin into the dorsal lateral surface of the left hind paw. For 30–40 minutes, then counted how many times each animal licked and bite. The reaction was bi-phasic, with the early phase being indicated by the initial nociceptive response (0–5 minutes after formalin injection) and the late phase being shown by (15–30 minutes). In a huge beaker, this experiment was conducted [32, 33].

*(iv) Anti-ulcer activity*. This activity was done by using two models which are described as following:

*(iv-a) Ethanol induced acute gastric ulcer model*. In this study design we have used five groups of animals which were chosen at random comprising of five animals in each group. The details of the group were as:

Group I: The control group received a vehicle (distilled water (10 mL/kg).

Group II: Standard (Omeprazole 20 mg/kg)

Group III: Ethanolic group (treated with distilled water 1 mL/100g of animal)

Group IV: Test group (leaves methanolic extract of *E*. *spinosa* 250 mg/kg body wt.)

Group V: Test group (leaves methanolic extract of *E*. *spinosa* 500 mg/kg body wt.

All groups fasted for 24 hours, but could stay hydrated. They were deprived of water for two hours after starting the trial. The goal of fasting was to prevent gastrointestinal reactions to treatments. Orally, each rat was given a pre-treatment based on weight and group. A 24-hour methanolic extract of *Emex spinosa* leaf (250 and 500 mg/kg, p.o.) was administered to the treatment groups, while the standard group received omeprazole dose at rate of 20 mg/kg, p.o. The negative control group received distilled water only. And after 30 minutes, all groups except the normal group were given 100% ethanol (1 mL/100 g). The animals were subsequently killed with anesthesia method using excessive chloroform or diethyl ether in CO_2_ chambers and their stomachs were removed. To assess gastric volume, total acidity, mucosa weight, pH and stomachs were split open along the greater curvature. The stomachs were then gently cleansed with saline solution and examined on a wax plate as previously cited protocol [34].

*(iv-b) Aspirin-induced chronic gastric ulcer*. According to Choi *et al*., (2010); the animals were fasted for 24 hours before being employed in the experiment [35]. They were dehydrated for two hours before the experiment. Each rat was given a pre-treatment orally, based on weight and group. The treatment group received 250 or 500 mg/kg of methanolic *Emex spinosa* leaf extract whereas the control group received omeprazole (20 mg/Kg). The negative control group got cars (distilled water) only. After a 24-hour fast, 30 minutes after extract treatment, the rats were given aspirin 250 mg/kg body weight. These treatments were given once a day for 14 days. At 15 days, all animals were starved for 24 hours but had free access to water and were separated into cages with one rat per cage. The animals were killed with use of excessive chloroform or diethyl ether in a CO_2_ environment to make them completely sense-less to make painless at time of sacrifice or slaughtering and their stomachs were removed very carefully for further experimental trials. To assess gastric volume, total acidity, mucosa weight and pH, stomachs were split open along the greater curvature. The stomachs were then gently cleansed with saline solution and examined on a wax plate [35].

### Parameters of gastric ulcer

#### Microscopic and macroscopic evaluation

The stomach was flushed with normal saline, and the changes in the inner walls of the stomach were examined macroscopically with a magnifying glass and a dissecting microscope. The number of lesions, red spots and hemorrhagic streaks is reported and used to calculate the ulcer index. The photos were taken with a smartphone camera [36].

#### Ulcer scoring

The ulcers were assigned ratings depending on their severity as stated in protocol of previous study [37].

0.0 = normal stomach or no ulcer

0.5 = red coloration or reddish mucosa

1.5 = hemorrhagic streaks

2.0 = deep ulcer

3.0 = perforations

#### Ulcer index

The method of Gul *et al*., was used to measure the ulcer index with some modifications [38].

*Ulcer index* = *(UN+US+UP)* × 10^−1^

UN = average no. of ulcer per animals

US = average of severity score

UP = % of animal with ulcer

#### Percentage protection

The following formula was used to measure the percentage of defence [38].


$$\%\;protection\;=\frac{ulcer\;index\;of\;treated\;group-ulcer\;index\;of\;control\;group\;}{ulcer\;index\;of\;treated\;group\;}\times 100$$

#### Determination of pH and gastric volume

The cardiac end of the stomach was dissected out and its contents drained into a falcon tube and after centrifugation at 2000 rpm for 10 minutes, take supernatant the volume of gastrointestinal juice was determined by previous researcher [39]. An aliquot part of the supernatant was taken and the pH of the fluid was measured using a digital pH meter as per cited protocol [40].

#### Determination of total acidity

Fill a vial of (one) 1 mL containing gastric juice. As an indication, use two drops of phenolphthalein. It was titrated with 0.01 N NaOH until a persistent pink colour was detected as the end stage. It was estimated how much 0.01 N NaOH was consumed. The following formula translates the total acidity into mEq/L [41, 42].


$$Total\;acidity=\frac{Volume\;of\;NaOH\;used\times Normality\;of\;NaOH\;used\times 100mEq/liter}{0.1}$$

#### Determination of mucosa weight

The aim of this portion of the experiment was to determine the weight of the mucosa layer that prevents lesions from developing. In this protocol procedure of Wahyuni et al., (2022) was followed [43]. During the stomach dissection, the contents were taken and placed in centrifuge tubes, which were centrifuged for 10 minutes at 1000 rpm. Centrifugation was used to isolate the mucosa from the stomach acid. The supernatant was used in a pH test before being removed, and the sediment (mucosa) was held in a falcon tube and weighed with a weight balance to determine the mucosa weight.

#### Histological investigation

The stomachs were fixed for 1 to 4 days with a 10% buffered formalin solution. After that, the stomachs were trimmed and covered in cold paper before being placed in the container. The stomachs were processed using an integrated tissue processing unit. The biopsied stomachs were then fixed in paraffin wax and cut into 5 micrometer tissue pieces using a rotary microtome. Finally, the collected tissue parts were stained with hematoxylin and eosin and examined under a light microscope to detect the presence of any histological alterations such as edema, necrosis, hemorrhage, and congestion in the gastric lesion and mucosa layer. Under 100 X magnification, the obtained thin section was examined [44].

### Statistical analysis

Results were expressed as Mean± SEM (standard error mean), one-way ANOVA one way analysis coupled with student ‘*t*’ test, LSD, were consider Statistically significant at p< 0.05*, 0.01** and 0.001*** as per protocol used in Ribeiro et al., [45].

## Results and discussion

### Phytochemical analysis

**Fig 1:**
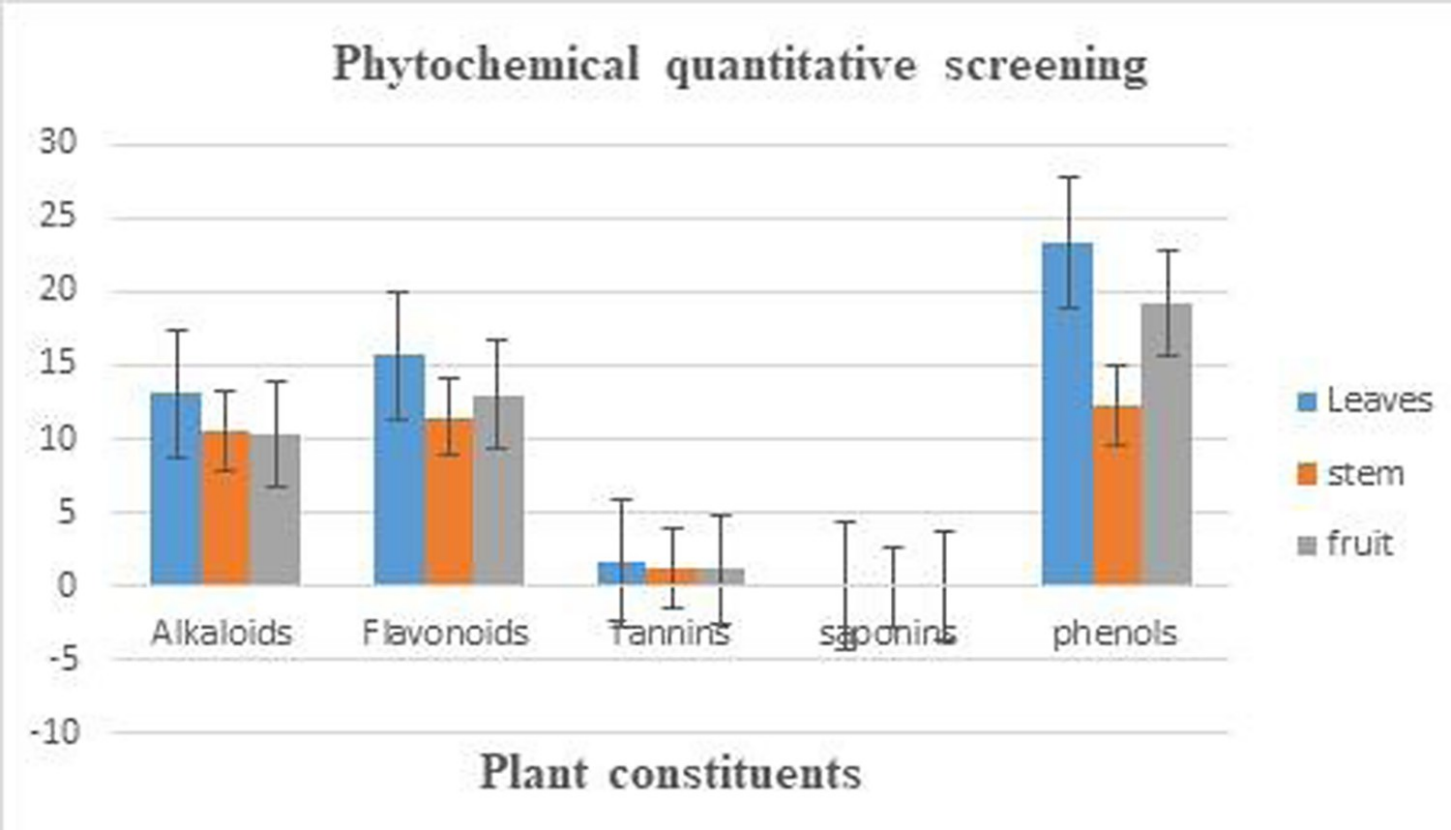
Graphical representation of phytochemical quantitative analysis of leaf, stem and fruit of *Emex spinosa*.

The phytochemical qualitative and quantitative examination indicated the existence of various abundant secondary metabolites in all three plant parts. The existence of carbohydrates, pholabatannis, phytosterol, alkaloids, flavonoids, phenols, tannins, Glycosides, proteins, steroids and triterpenoids were present in the leaf, fruit and stem whereas were saponins in all three parts of and glycosides were absent in fruit and stem of experimental plant *E*. *spinosa* (Table 1, Fig 1) and similar findings have been reported in the previous past research work in which it was stated that plant has many phytoconstituents and these phytochemicals were responsible for the efficacy as medicine [46].

**Table 1:** Phytochemical qualitative analysis of *E*. *spinosa* MeOH extract from Mirpur AJK, Pakistan.

Phytochemical constituents	Phytochemical tests	Leaf	Fruit	Stem
**Carbohydrates**	Benedict’s Analysis	**+++**	**_**	**_**
	Molisch’s Analysis	**+++**	**_**	**_**
**Phlobatannins**	Hydrochloric acid test	**++**	**_**	**+**
**Phytosterol**	Liebermann Burchard analysis	**+++**	**_**	**_**
**Saponin**	Forth analysis	**_**	**_**	**_**
**Alkaloids**	Drangendroff’s analysis	**+++**	**++**	**++**
	Mayer’s analysis	**+++**	**++**	**+++**
**Flavonoids**	Sulphuric acid reaction	**+++**	**+++**	**+++**
	Ferric chloride reaction	**+++**	**+++**	**+++**
**Steroids and Terpenoids**	Salwoski analysis	**++**	**+**	**++**
	Liebermann- Burchard analysis	**++**	**+**	**++**
**Tannins**	Lead acetate reaction	**+**	**+**	**+**
	Ferric chloride reaction	**+**	**+**	**+**
**Glycosides**	Keller-Killani analysis	**+++**	**_**	**_**
	Borntrager’s analysis	**+++**	**_**	**_**
**Protein**	Xanthoproteic Analysis	**++**	**_**	**_**
	Millon’s analysis	**++**	**_**	**_**

**Key:** -: Absent; + present in minute conc.;++ present in fair conc.;+++ present in signficamt conc.

The phytochemical quantitative analysis of methanolic extract revealed that alkaloids were present in the highest quantity in leaf extract 13.03±0.38 μg, and lowest quantity in stem extract 10.36±0.26 μg. Flavonoids were quantified in highest amount in leaf extract 15.63±0.93 μg and the lowest in stem extract11.5±0.57 μg. Tannins were present as the highest amount in leaf extract 1.59±0.05 μg and the lowest in stem 1.15±0.07 μg. The phenols were present highest amount in leaf extract 23.25±0.5 and lowest in stem 12.22±0.1 μg whereas saponin were absent in all three samples of *E*. *spinosa* (Table 1; Fig 1). The results were compared with the previous work which was based on evaluating the phytochemical evaluation of different parts of *Himalrandia tetrasperma* which is near relative and medicinal plants [47]. Albeit similar and congruent results outcomes were also reported by the Ajaib et al., (2010) and Ishtiaq et al., 2021 [48–50].

### Antidiabetic analysis

The findings of anti-diabetic activity of methanolic extract of leaf, fruit and stem of *Emex spinosa* revealed that in acute study after a single dose of 500 mg/kg extracts the blood glucose level was after 2 hrs mg/dl were 367±51.09958^NS^, 416±59.79548^NS^, 437.5±61.96437^NS^ and after 4 hrs 351.75±88.27644^NS^ mg/dl, 448.25±25.64948^NS^ mg/dl, 445.25±27.07205^NS^ mg/dl and after 6 hrs 354.5±92.70428^NS^, 442±24.60691^NS^, a440±33.16625^NS^ mg/dl, respectively there was no significant reducing diabetic effect (Table 2). In subacute study after giving (leaf, stem and leaf) extracts dose (500 mg/kg), the readings of body glucose level (BGL) after 7 days revealed that there was no decease in blood glucose levels in all three treatment groups (leaf, fruit and stem), which showed non-significant results as compared to normal control and standard glibenclamide 10 mg/kg group (Table 2), the results were compared with the work of Venkatesh *et al*., who evaluated the anti-diabetic activity of flowers of *Hibiscus rosa-chinensis* [47]. Fruit extract was verified via Oral Glucose tolerance at dose 500 mg/kg in alloxan treated rat. Findings showed that after 30 mins readings were 122.5±5.634714 ^NS^, after1hr 126±4.143268 ^NS^, 2hr 136.25±7.341378 ^NS^, 3hr 134.75±7.364046^NS^, 4hr 136±7.141428^NS^, and 6hr 142±6.670832^NS^, respectively; as compared to acarbose 4.28 mg/kg body weight results was compared with of Sikarwar et al., [49]. Results from oral glucose tolerance *test* (OGT) test showed that due to a lack of some biological products such as saponins all of three parts were failed to overcome diabetes (Table 2). Analgesic activity of leaf, fruit and stem of *E*. *spinosa* were investigated by applying hot plate, tail flick and formalin paw licking method. In hot plate method the maximum results were observed with 250 mg/kg in the 2.5 hours of study. The leaf extract showed a 40.41±2.73 latency time and the fruit with a 36.77±2.41, and the stem with a 27.85±3.09, which was comparable to the standard drug Aspirin, *i*.*e*., 47.5±0.57. (Tables 2 and 3). The respective plant extracts at 250 mg/kg showed a gradual rise in latency time when compared to the control. All three components of *E*. *spinosa* perform well in hot plate techniques. The overall results were found to be statistically significant (P < 0.05 and p < 0.01). These results were compared with the work of Ajaib et al., who claimed the antimicrobial and antidiabetic evaluation of *Dicliptera bupleuroides* Nees [51].

**Table 2:** Effects of *E*. *spinosa* leaf, stem, and fruit methanolic extract on blood glucose levels in alloxan-induced diabetic rats after a single oral dose (*blood glucose level measured in mg/dl*).

Treatments	Fasting dl/mg	After Alloxan dl/mg	After 2hrs with dose of leaf extract	After 4hrs with dose of leaf extract	After 6hrs with dose of leaf extract
**(Nc)**	85±1.99	105.25±0.71	86.27±1.44	84.68±1.28	85.62±1.10
**(Dc)**	95.12±1.69***	178.74±1.49***	181.04±1.46***	191.31±1.42**	193.77±1.26**
**(Tc)**	92±1.81***	189.18±1.99***	178.39±1.99***	171.16±2.12***	157.66±2.04***
**Leaf part 500mg/kg (T1)**	91.25±1.1388^NS^	355.5±71.25249^NS^	367±51.09958^NS^	351.75±88.27644^NS^	354.5±92.70428^NS^
**Fruit part 500mg/kg (T2)**	91.25±1.1388^NS^	430.75±48.2759^NS^	416±59.79548^NS^	448.25±25.64948^NS^	442±24.60691^NS^
**Stem part 500mg/kg (T3)**	90.5±1.6007^NS^	398.75±65.45912^NS^	437.5±61.96437^NS^	445.25±27.07205^NS^	440±33.16625^NS^

Values are reported as Mean ± Standard Error Mean (n = 4), and one-way ANOVA was used to do the study. NS = not significant

* = low significant

** = moderate significant

*** highly significant.

**Table 3:** Oral glucose tolerance test for antidiabetic activity of fruit of *E*. *spinosa* from AJK, Pakistan (blood glucose level mg/dl).

Groups and Treatments	Fasting mg/dl	After 30 min	1hrs	1.5hr	2hrs	3hr	4h	5hr	6hr
A.	87.75±1.1288	129±1.6101	134.75±1.6723	140.25±1.2830	144.25±1.0815	123±1.1626	130.75±1.1934	111.75±0.8936	102.75±1.83
B.	155.65±1.6335	204.75±2.4076	222.25±1.4206	230±1.4576	236.65±0.9501	223.25±1.1525	202.75±1.083	183.5±1.0307	151.25±1.1328
C.	97.24±1.4144	109±1.88708*	124.5±1.4261**	135.25±1.0825*	142±0.49**	136.65±1.0725*	124.65±1.0724*	112.75±1.9495**	102.75±1.18575**
D.	99.75±2.1360009	122.5±5.634714 ^NS^	126±4.143268*	112±12.06234*	136.25±7.341378	134.75±7.364046*	136±7.141428 ^NS^	138.25±7.284401 ^NS^	142±6.670832^NS^

Values are reported as Mean ± Standard Error Mean (n = 4 animals per group), and one-way ANOVA was used to do the study. NS = not significant

* = low significant

** = moderate significant

*** highly significant.

### Antiinflammatory analysis

**Fig 5:**
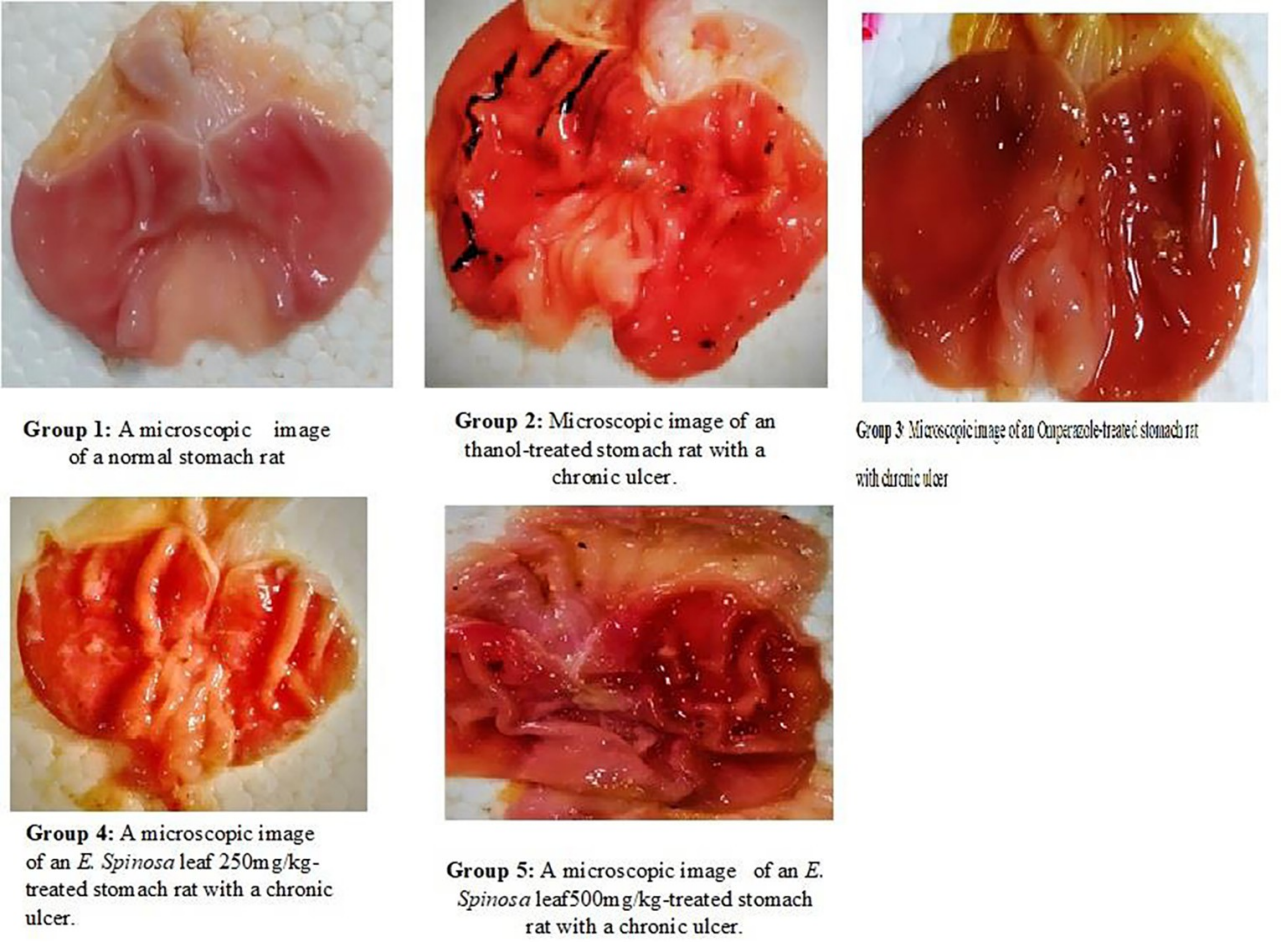
Microscopic evaluation of chronic aspirin -induced gastric ulcer group 1–5 represents the numbers of lesions, red spots, and hemorrhagic streaks.

The pain reaction time (PRT) at 0 hr from 8.79 ± 1.31, 7.87±1.16, and 6.76±0.99 shown by leaves, fruit, and stem extract of 250 mg/kg were increased at 2 hrs. For leaf, fruit, and stem were 19.27±0.43, 16.41±0.82, and 12.47±2.25 as compared to aspirin, *i*.*e*., 24.3 ±0.48. Aspirin prolongs the PRT relative to the control group. The mean PRT increased somewhat to 250 mg/kg of leaf, fruit, and stem extracts. The results of the tail flick method showed that leaf and fruit extracts showed significant results, whilst stems showed promising results. When compared to the control group, the tail flick revealed that the leaf, fruit, and stem extracts at a dose of 250 mg/kg, as well as the drug Aspirin, significantly increased the pain response (Table 4; Fig 5). The overall results were found to be statistically significant (P < 0.05 and p < 0.01). These results were compared with the work of Ajaib et al., who did evaluation by using phytochemical, anti-inflammatory and analgesic Screening of an ethnomedicinal plant *Engelhardia colebrookiana* Lindl [52, 53].

**Table 4:** The Hot plate technique was used to test the analgesic efficacy of a methanolic extract of *E*. *spinosa* leaf, fruit, and stem at a concentration of 250 mg/kg.

Time	Leaf (250 mg/kg)	Fruit (250 mg/kg)	Stem (250 mg/kg)	Aspirin (300 mg/kg)	Control
0h	17.31±2.15	10.49±0.62	14.08±2.37	11.0±0.04	12.5±0.57
0.5h	36.18±4.07	31.13± 4.76	22.31±2.10	14.1±0.05	13.5±0.27
1h	35.65±4.11	32.45±3.18	22.6±4.62	16.5±0.13	16.0±0.54
1.5h	36.61±8.00	31.79±4.04	28.15±4.96	21.2±0.11	14.7±0.82
2h	33.53±3.29	32.37±4.85	29.35±3.93	21.1±0.07	14.0±1.64
2.5h	40.41±2.73	36.77±2.41	27.85±3.09	27.5±0.57	15.0±0.99
3h	28.26±6.22	28.1±3.93	25.9±3.14	19.7±0.35	12.1±0.36
3.5h	25.51±5.70	19.62±1.82	17.34±1.75	19.1±0.57	11.5±0.30
4h	20.24±4.37	18.38±1.76	16.15±1.75	20.7±0.77	10.7±0.36
4.5h	19.53±4.26	18.16±0.89	15.22±1.37	16.8±0.78	10.3±0.23

Results were expressed as Mean± SEM (standard error mean), n = 5. P < 0.05 and P> 0.05. Statistically significant from control and standard drug.

#### Analgesic efficacy analysis

**Fig 2:**
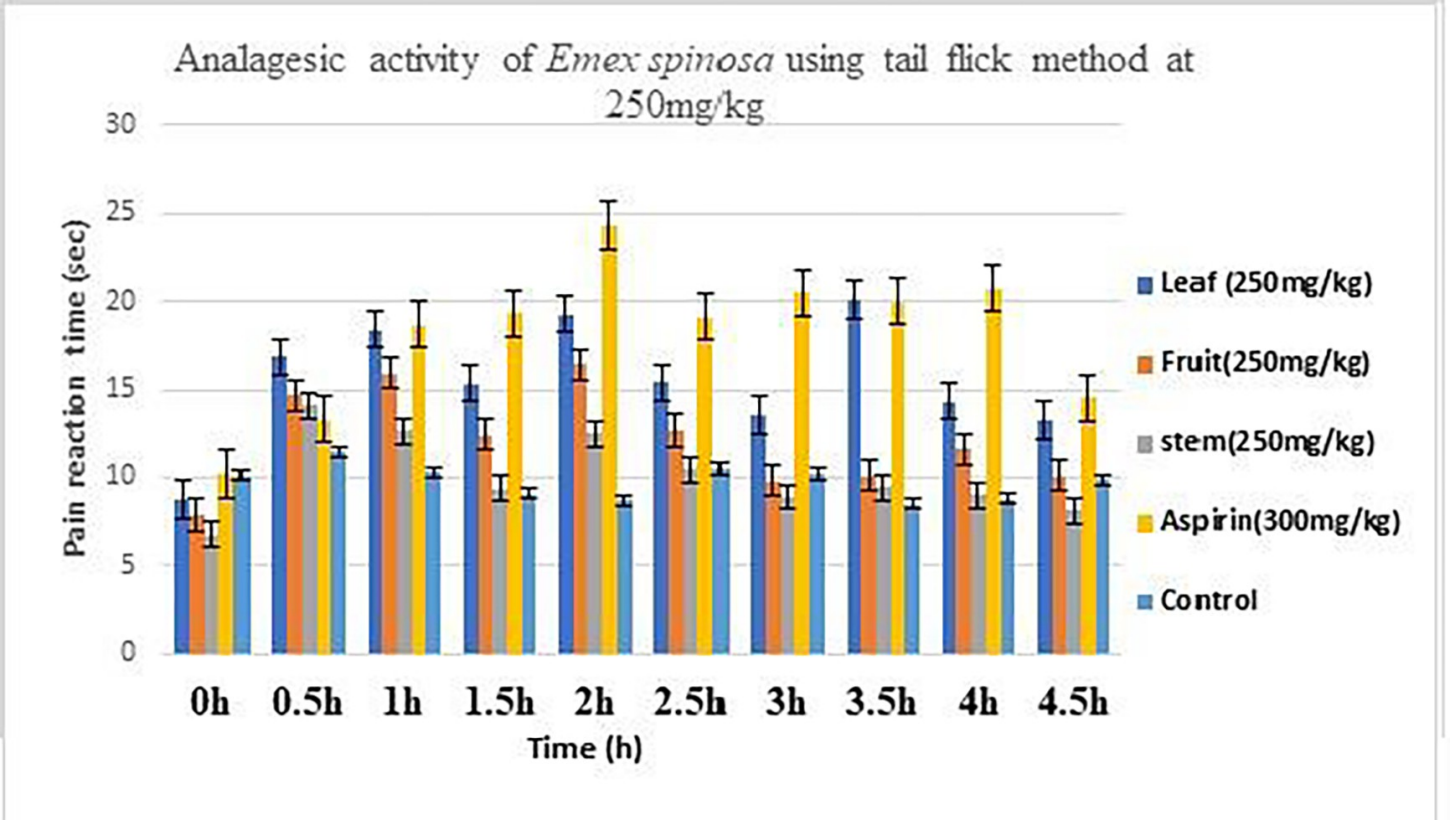
Analgesic activity of *E*. *spinosa* leaf, fruit, and stem collected from Bhimber AJK.

**Fig 3:**
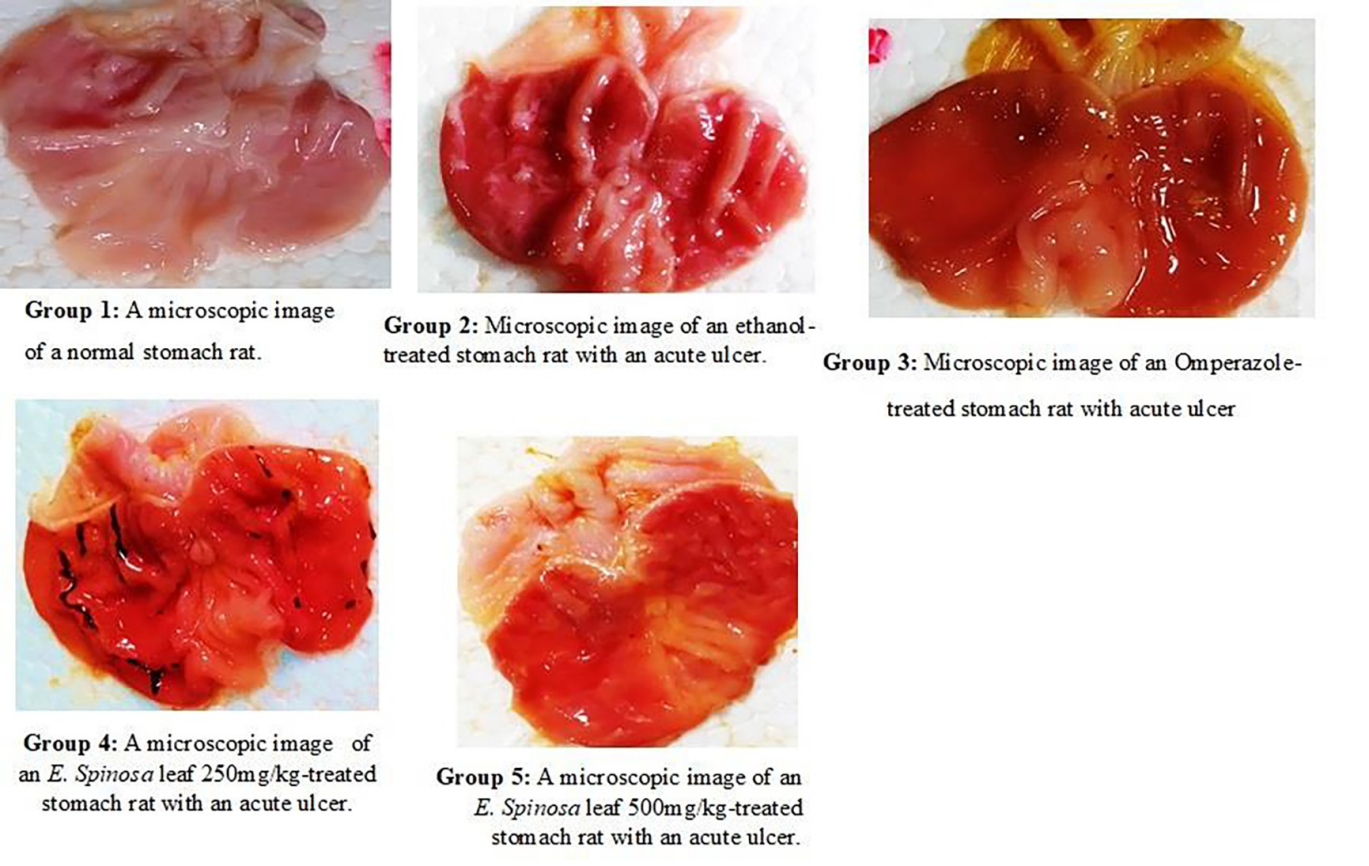
Microscopic evaluation of acute ethanolic-induced gastric ulcer group 1–5 represents the numbers of lesions, red spots, and hemorrhagic streaks.

**Fig 4:**
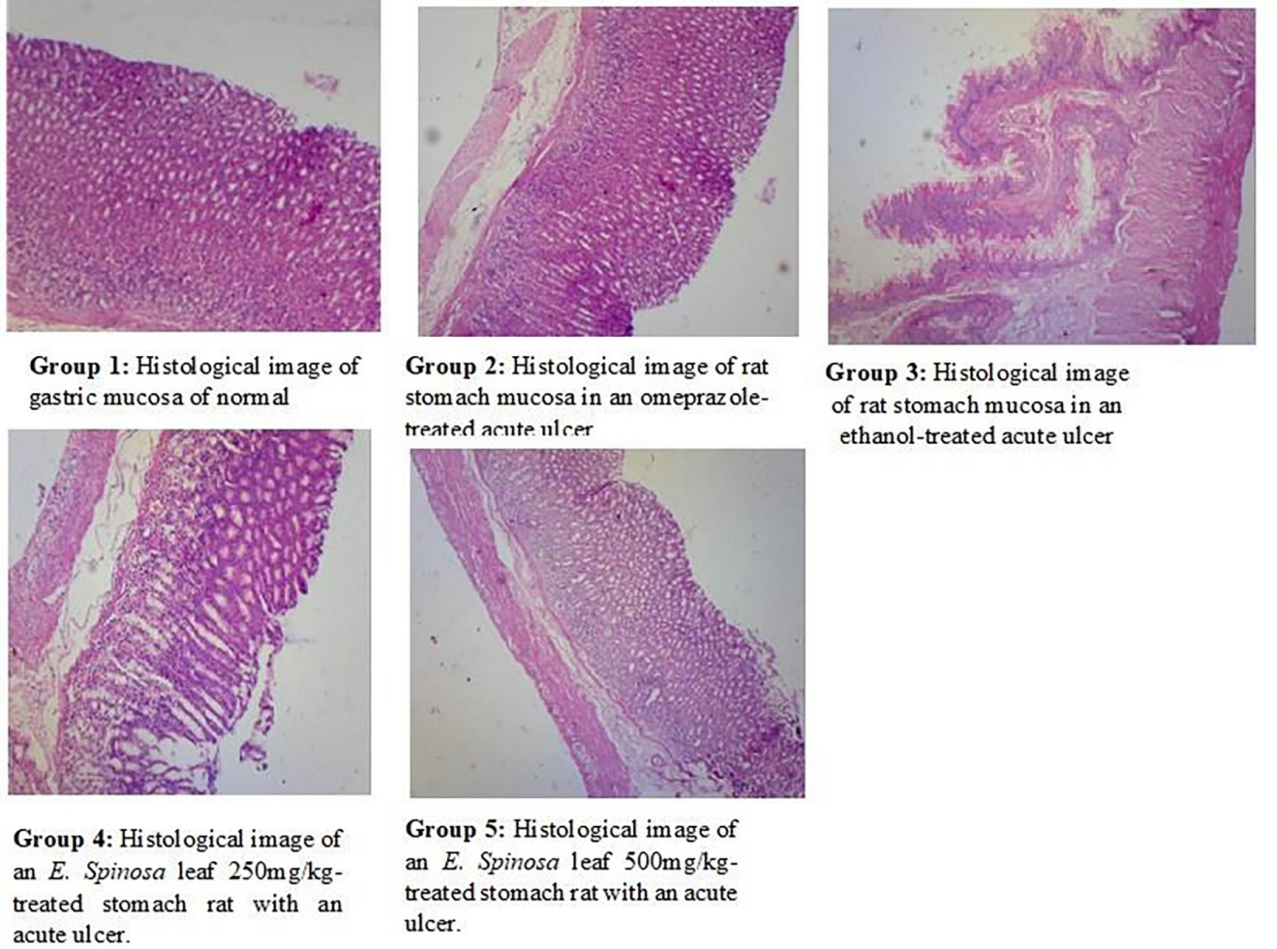
An ethanol-induced gastric Group 1–5 histological abnormalities such as edema, necrosis, hemorrhage, and congestion in the gastric lesion and mucosa layer. The thin section was examined at 100X magnification.

Results of Formalin Pain Induced (FPI) method are shown in tabular form (Table 5). In both phases, all oral extract doses reduced paw shaking, licking, and biting of the formalin-injected paw. Plant extracts from the leaves, fruit, and stem had a strong inhibitory effect on both phases of formalin-induced pain. The plant demonstrated substantial results at 250 mg/kg. Total paw licking and biting decrease with 250 mg/kg extract. The results of formalin paw licking and biting revealed that at 10 minutes, leaf, fruit, and stem showed significant results of 3.06±0.93, 3.66±0.24 and 3.97±0.77, respectively, as compared to the non-extract group 7.36 ±2.01 (Tables 5 and 6, Fig 2). At 15 minutes, 2.76±0.50, 2.81±0.87, 2.82±0.60, respectively, as compared to the non-extract group 6.99**±**0.54 (Figs 3 and 4). At 20 minutes 2.11±0.51, 2.15 ±0.79, 2.80±0.82, respectively, as compared to aspirin **(**2.12 ±0.77) and non-extract group 5.62±1.45 (Fig 2). Values become decrease with time. In comparison to the control group, the respective plant components showed significant (P**<** 0.05 and p < 0.01**)** findings, and this herb reduced the rats’ nociceptive response. According to the findings, the plant has a significant antinociceptive impact. The findings were compared with work of Sanmugapriya and Venkataraman, (2007) who did investigated the analgesic activity of ethanolic root extract of *Croton zambesicus* [51].

**Table 5:** The tail flick technique was used to test the analgesic efficacy of a methanolic extract of *E*. *spinosa* leaf, fruit, and stem at a concentration of 250 mg/kg.

Time	Leaf (250mg/kg)	Fruit (250mg/kg)	Stem (250mg/kg)	Aspirin (300mg/kg)	Control
0h	8.79±1.31	7.87±1.16	6.76±0.99	10.2±0.37	10.1±0.31
0.5h	16.84±0.65	14.66±2.00	14.12±2.09	13.3±0.03	11.5±0.02
1h	18.4±0.93	15.91±1.87	12.64±2.15	18.7±0.23	10.3±0.03
1.5h	15.36±2.04	12.41±2.28	9.39±1.74	19.3±0.12	9.1 ±0.01
2h	19.27±0.43	16.41±0.82	12.47±2.25	24.3±0.48	8.7±0.41
2.5h	15.39±1.53	12.66±1.83	10.49±2.22	19.1±0.05	10.5±0.45
3h	13.58±2.35	9.79±2.03	8.9±2.61	20.5±0.35	10.2±0.72
3.5h	20.06±1.70	10.13±2.56	9.47±1.98	20.0±0.58	8.5±0.03
4h	14.34±1.54	11.64±1.56	9.01±2.57	20.7±0.77	8.8±0.1
4.5h	13.26±1.81	10.08±2.28	8.14±2.46	14.5±0.4	9.9±0.3

Results were expressed as Mean± SEM (standard error mean), n = 5. P < 0.05 were considered statistically significant

**Table 6:** The formalin—induced hind paw licking in mice technique was used to test the analgesic efficacy of a methanolic extract of *E*. *spinosa* leaf, fruit, and stem at a concentration of 250 mg/kg.

Time	Leaf (250mg/kg)	Fruit (250mg/kg)	Stem (250mg/kg)	Aspirin (100mg/kg)	Without extract (after formalin induction)
5	4.2 ±0.38	4.91±1.16	5.4±0.43	8±1.00	7.06±0.89
10	3.06±0.93	3.66±0.24	3.97±0.77	3.77±1.22	7.36 ±2.01
15	2.76±0.50	2.81±0.87	2.82±0.60	2.01 ±0.58	6.99±0.54
20	2.11±0.51	2.15 ±0.79	2.80±0.82	2.12 ±0.77	5.62±1.45
25	2.84±1.02	3.05±1.27	2.81±0.82	1.12 ±0.14	5.87±1.01
30	2.65±1.20	2.86 ±0.50	3.65±0.39	0.49±0.93	2.22±1.56

Results were expressed as Mean± SEM (standard error mean), n = 5. P < 0.05 were considered statistically significant.

### Antiulcer activity

Ulcers is formed stomach or intestines when the stomach or duodenal lining’s normal defence and healing systems are interrupted, leaving the lining more exposed to acid damage. It is a sore in the stomach, small intestine, or throat. Inhibiting prostaglandin synthase (PG) synthesis or increasing mucus production are two ways to reduce stomach acid secretion [41, 53–55]. According to Lakshmi, an imbalance between defensive and aggressive components like as acid, *H*. *pylori* and pepsin causes peptic ulcer [56]. Various chemicals induce gastrointestinal injury. Toxic effects of aspirin include mucosal damage, acid release, and back diffusion of H + ions [57]. The ethanol-induced stomach mucosal injury is complex. Its rapid penetration into the stomach mucosa promotes increased mucosal permeability and vasoactive substance release, causing vascular and gastrointestinal damage [58, 59].

The current study investigated the effects of *E*. *spinosa* leaf methanolic extract on acute ulcer activity. The ability of *E*. *spinosa* leaf extract to inhibit ulcer formation was also studied. The inhibition %, ulcer index, gastric volume, mucus weight, and stomach pH content were determined using key guidelines from Fitzpatrick et al [41, 60]. We created two groups of plant leaf methanolic extract (250 mg/kg and 500 mg/kg) in addition to the negative and positive controls. The negative control was to examine if plant extracts differed in their ability to inhibit stomach ulcers. The positive control, omeprazole, demonstrated antiulcerogenic activity.

**Fig 6:**
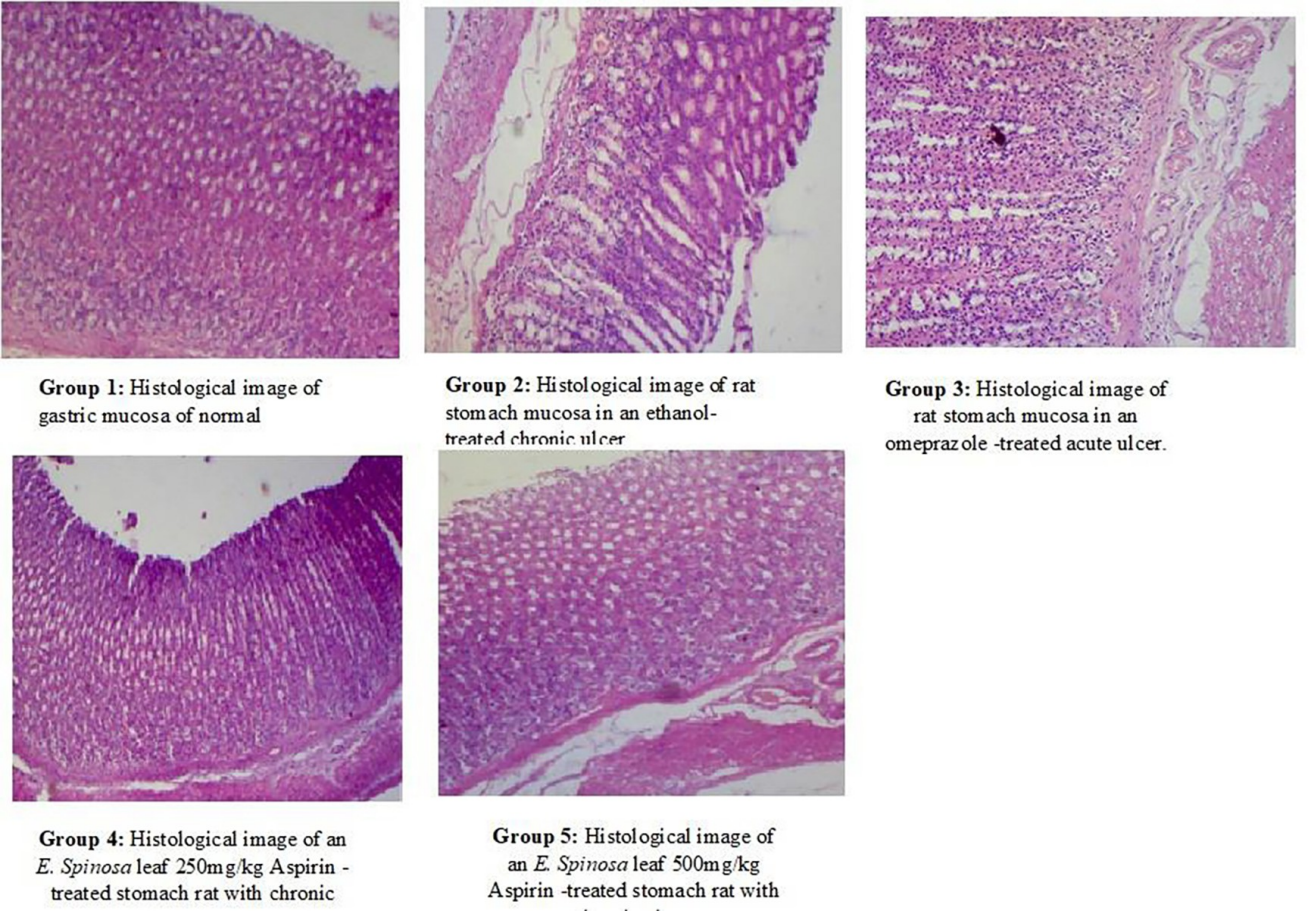
An aspirin-induced gastric Group 1–5 histological abnormalities such as edema, necrosis, hemorrhage, and congestion in the gastric lesion and mucosa layer. The thin section was examined at 100X magnification.

A microscopic examination of stomach revealed that there was no ulcer in the normal group and significant ulcer streaks and patches were observed in the stomachs of animals pre-treated with ethanol in acute ethanol-induced gastric ulcer. However, the control medication, omeprazole, caused minor ulcers and primed the stomach wall against ethanol-induced ulcers. Similarly, *E*. *spinosa* leaf extracts at 250 mg/kg and 500mg/kg revealed tiny ulcer patches and strikes, indicating a protective effect against ethanol-induced damage to the mucosal wall (Fig 3). Gastric volume, pH and total acidity in acute ethanol-induced gastric ulcer gastric ulcer, in leaf extracts at a dose of 250mg/kg were 1.36±0.11**, 3.64±0.14***, and 45.58±5.95***, respectively (Figs 4–6), and at dose of 500mg/kg, the gastric volume was 0.96±0.15***, the pH of gastric content was 4.12±0.12***, and the total acidity was 40.83±5.82*** (Table 7) and ulcer index, mucosa weight, % inhibition at dose 250mg/kg were 1.005***, 0.25±0.020*** and 59.55***, respectively (Figs 4–6). At dose 500 mg/kg were 1.321***, 0.375±0.242*** and 75.59***, respectively (Tables 9 and 10). The gastric volume of ethanol-treated rats was greater than that of control rats. A gastric ulcer causes more gastric juice. However, *E*. *spinosa* leaf 250 mg/kg and 500 mg/kg sought to lower stomach volume while also showing gasteroprotective properties. Similarly, ethanol-treated groups had lower pH than control groups. Less ethanol and greater *E*. *spinosa* leaf elevated pH than ethanol, indicating protection of the stomach mucosa wall. The ethanol-treated group’s stomach juice had a higher overall acidity. More acidity means more stomach ulcers. The 250 mg/kg and 500 mg/kg *E*. *spinosa* leaves lowered overall acidity, indicating protection of the stomach mucosa wall. The ulcer index increased following ethanol treatment. The ulcer index of control group seemed to be dropped and it was found that omeprazole worked better and similar results were narrated by Hammad et al., [61]. Similarly, compared to the ethanolic group, *E*. *spinosa* leaf 250 mg/kg and 500 mg/kg showed a substantial reduction in ulcer index. The ethanol-treated groups had less mucosa than the control groups. Less ethanol and more *E*. *spinosa* leaf enhanced mucosa content than ethanol. Compared to the ethanolic group, two distinct dosages of *E*. *spinosa* showed the highest inhibitory protection against ethanol-generated stomach ulcers in experimental rats. These doses decreased stomach volume, raised pH, and increased total, indicating possible antiulcer drugs. These data reveal that *E*. *spinosa* 500 mg/kg outperforms leaf methanolic extract 250 mg/kg. The ethanol-treated mice’s stomach mucosa was severely damaged histologically. Congestion effusion, apoptotic cells other groups observed similar characteristics, although with lower ethanol concentrations [62].

**Table 7:** Effect of different methanolic extracts of *E*. *spinosa* on gastric ulcer, gastric volume, pH, total acidity in acute ethanol-induced gastric ulcer.

Treatments	Dose(mg/kg) p.o.	Gastric Volume (ml)	pH of gastric content	Total acidity (mEq/L)
**Normal saline**	10ml/kg p.o.	1.2±0.1	3.81±0.11	28.33±2.03
**Ethanol**	10ml/kg p.o.	3.81± 0.23	2.54 ±0.12	98.17±3.82
**Omeprazole**	20mg/kg	1.80±0.29***	5.96±0.49***	32.33±2.80***
**Methanolic leave Extract**	250mg/kg	1.36±0.11**	3.64±0.14***	45.58±5.95***
**Methanolic leave Extract**	500mg/kg	0.96±0.15***	4.12±0.12***	40.83±5.82***

Results were expressed as Mean± SEM (standard error mean), n = 4. one-way ANOVA one way analysis coupled with student ‘t’ test, LSD, were consider Statistically significant at p< 0.05*, 0.01** and 0.001*** when compared to control group.

In Aspirin-induced gastric ulcer models, increased release of stomach acid and pepsin leads to autoimmunity, gastric mucosa digesting, and mucosal barrier disintegration [59, 63]. A decrease in bicarbonate and mucus output due to aspirin inhibition. Backflow of H+ causes serious gastrointestinal damage. Developing a protective layer over the stomach mucosa and increasing the mucosal barrier may explain the test drug’s protection in this model. Aspirin-induced stomach damage may be connected to free radical scavenging [59–63].

The normal group had no ulcers, but the animals pre-treated with aspirin-induced gastric ulcer had large ulcer streaks and patches. It created small ulcers and primed the stomach wall against aspirin-induced ulcers. To prevent the mucosal wall against aspirin-induced damage, 250mg/kg and 500mg/kg *E*. *spinosa* leaf extracts showed small ulcer patches and strikes (Tables 8–10; Fig 5). Gastric volume, pH, total acidity in acute ethanol-induced gastric ulcer gastric ulcer, in leaf extracts at a dose of 250 mg/kg were 1.36±0.11**, 3.64±0.14***, and 50.14±0.28** respectively (Figs 3–6), and at dose of 500mg/kg, the gastric volume was 1.28±0.17***, the pH of gastric content was 4.3±0.122*** and the total acidity was 30.62±0.26*** And ulcer index, mucosa weight, % inhibition at dose 250mg/kg were 3.11**, 0.124±0.036** and 60.52** respectively (Figs 4–6). At dose 500mg/kg were 1.1916***, 0.252±0.042*** and 79.62***, respectively (Tables 9 and 10). Results were considered statistically, significant at p< 0.05*, 0.01** and 0.001*** when compared to control group. Gastric juice characteristics reveal a drug’s antiulcer activity [Table 10]. The stomach capacity of ethanol-treated mice was high, but control and treatment group drugs reduced gastric volume to normal animal levels. Less than half of the ethanolic group’s stomach capacity was lost at 500mg/kg [Fig 6]. The control drugs all lowered stomach volume. Similarly, ethanol-treated groups had a lower pH than control groups. This means that the *E*. *spinosa* leaf 250 mg/kg and 500 mg/kg groups elevated pH more than the ethanol group. Among the experimental groups, *E*. *spinosa* leaf extract 500 mg/kg showed the largest pH elevation. The ethanol-treated group’s stomach acidity was higher than normal. A higher overall acidity indicates a stomach ulcer. Overall acidity was reduced by methanolic *E*. *spinosa* leaf extract at 250 mg/kg and 500 mg/kg, protecting the stomach mucosa wall. The 500 mg/kg *E*. *spinosa* leaf extract significantly reduced total acidity in the experimental group. As a consequence, both control and experimental drugs reduced stomach volume, pH, and overall acidity, indicating ulcer-inhibitory potential. The ethanol-treated group’s ulcer index was elevated due to high ulcer grade. Thus, *E*. *spinosa* 500 mg/kg had a lower ulcer index than *E*. *spinosa* 250 mg/kg. Similarly, compared to the ethanolic group, E. *spinosa* leaf 250 mg/kg and 500 mg/kg showed a substantial reduction in ulcer index [Tables 9 and 10; Figs 5 and 6]. The ethanol-treated groups had less mucosa than the control groups. Less ethanol and more *E*. *spinosa* leaf enhanced mucosa content than ethanol. Similarly, 500 mg/kg *E*. *spinosa* leaf extract had more mucosa than 250 mg/kg omeprazole or the control group. Compared to the ethanolic group, two distinct dosages of *E*. *spinosa* showed the highest inhibitory protection against ethanol generated stomach ulcers in experimental rats. Similarly, *E*. *spinosa* leaf extract at 500 mg/kg demonstrated superior inhibition than omeprazole. These data reveal that *E*. *spinosa* 500 mg/kg outperforms leaf methanolic extract 250 mg/kg. The ethanol-treated mice’s stomach mucosa was severely damaged histologically [Figs 5 and 6]. Congestion effusion, apoptotic cells other groups observed similar characteristics, although with lower ethanol concentrations. Results were compared with Gamal et al., [63]. Evaluating the anti-ulcer activity of methanolic extract of *Jatropha curcas* L. on Aspirin-Induced Gastric Lesions in wistar Rat was cited in previous works and our results were congruent with those proving that the selected medicinal plants has very good potential for novel drug discovery [60–63].

**Table 8:** Effect of different methanolic extracts of *E*. *spinosa* on ulcer index, mucosa weight, % inhibition in acute ethanol-induced gastric ulcer.

Treatments	Dose(mg/kg) p.o.	Ulcer index	Moussa weight (g)	%Inhibition of ulcer
**Normal saline**	10ml/kg p.o.	0	0.512±0.015	0
**Ethanol**	10ml/kg p.o.	11.36	0.025±0.012	0
**Omeprazole**	20mg/kg	3.46	0.401±0.255***	69.56***
**Methanolic leaf Extract**	250mg/kg	1.005***	0.25±0.020***	59.55***
**Methanolic leaf Extract**	500mg/kg	1.321***	0.375±0.242***	75.59***

**Table 9:** Effect of different methanolic extracts *E*. *spinosa* on gastric ulcer, gastric Volume, pH, and total acidity in chronic aspirin-induced gastric ulcer.

Treatments	Dose(mg/kg) p.o.	Gastric Volume (mL)	pH of gastric content	Total acidity (mEq/L)
**Normal saline**	10ml/kg	1.15±0.19	3.93±0.15	28.5±2.4
**Ethanol**	10ml/kg	2.88± 0.22	3.17±0.21	83.33±4.03
**Omeprazole**	20mg/kg	1.4 ± 0.26***	6.29±0.16***	40.17±3.91
**Methanolic** **leave Extract**	250mg/kg	1.36±0.11**	3.64±0.14**	50.14±0.28**
**Methanolic** **leave Extract**	500mg/kg	1.28±0.17***	4.3±0.122***	30.62±0.26***

Results were expressed as Mean± SEM (standard error mean), n = 4. one-way ANOVA one-way analysis coupled with student ‘t’ test, LSD, were consider statistically significant at p< 0.05*, 0.01** and 0.001*** when compared to control group.

**Table 10:** Effect of different methanolic extracts *E*. *spinosa* on gastric ulcer, pH, total acidity in chronic aspirin-induced gastric ulcer.

Treatments	Dose(mg/kg) p.o.	Ulcer index	Moussa weight (g)	%Inhibition of ulcer
**Normal saline**	10ml/kg	0	0.49±0.015	100
**Ethanol**	10ml/kg	10.8	0.025±0.02	0
**Omeprazole**	20mg/kg	5.22**	0.306±0.027***	69.56***
**Methanolic leaf Extract**	250mg/kg	3.11**	0.124±0.036**	60.52**
**Methanolic leaf Extract**	500mg/kg	1.1916***	0.252±0.042***	79.62***

Results were expressed as Mean± SEM (standard error mean), n = 4. one-way ANOVA way analysis coupled with student ‘t’ test, LSD, were consider Statistically significant at p< 0.05*, 0.01** and 0.001*** when compared to control group.

## Conclusion

The plant *E*. *spinosa* was found to possess good medicinal potential for curing diseases of diabetes, ulcer and analgesic in model rat experimental animals. This study reveals and confirms that this traditional ethnomedicinal plant (TEMP) has very pivotal role in daily life of human being dwelling in the study of Azad Jammu and Kashmir, Pakistan. This plant has powerful painkiller function is significant. More pharmacological research is required to assess *Emex spinosa* extract’s safety in humans and it is also potential of discovery of neo-drugs allopathic medicines by using dedicated analytical and pharmaceutical analysis protocols.
