# Expression of Concern: Analysis of antidiabetic, antiulcer and analgesic potential of traditional ethnomedicinal plant *Emex spinosa* (L.) Campd. from Azad Jammu and Kashmir

**DOI:** 10.1371/journal.pone.0351608

**Published:** 2026-06-15

**Authors:** The PLOS One Editors

The *PLOS One* Editors issue this Expression of Concern for this article [1] because of concerns identified about the peer review process. Readers are advised to interpret the article [1] with caution.
